# Fiber-Optic Fabry-Pérot Interferometers for Axial Force Sensing on the Tip of a Needle

**DOI:** 10.3390/s17010038

**Published:** 2016-12-26

**Authors:** Steven Beekmans, Thomas Lembrechts, John van den Dobbelsteen, Dennis van Gerwen

**Affiliations:** 1 Department of Physics and Astronomy and LaserLab Amsterdam, Vrije Universiteit Amsterdam, Amsterdam 1081 HV, The Netherlands; 2 Department of Biomechanical Engineering, Delft University of Technology, Delft 2628 CD, The Netherlands; thomas.lembrechts@gmail.com (T.L.); j.j.vandenDobbelsteen@tudelft.nl (J.v.d.D.); D.J.vanGerwen@tudelft.nl (D.v.G.)

**Keywords:** Fabry-Pérot interferometer, needle-tissue interaction force, low temperature cross-sensitivity, fiber optic force sensor, tip force sensing

## Abstract

A range of complex percutaneous procedures, such as biopsy or regional anesthesia, rely heavily on accurate needle insertion. Small variations in the mechanical properties of the pierced tissue can however cause deviations from the projected needle path and can thus result in inaccurate placement of the needle. Navigation of a rigid needle towards the target tissue is traditionally based on the surgeons capacity to interpret small variations in the needle insertion force. A more accurate measurement of these small force variations enables improvement in needle targeting, can potentially aid in enhancing force feedback in robotic needle placement and can provide valuable information on tissue-tool interaction. In this study we investigated several concepts for the design of a force sensor based on a fiber-optic Fabry-Pérot interferometer to measure needle-tissue interaction forces on the tip of a 18 G needle, where special attention was given to concepts for a sensor with (1), an intrinsic low cross-sensitivity to temperature and (2), elementary design and fabrication. Three concepts, using either a quartz capillary, an Invar capillary or a thin polyimide film as the force sensitive element were prototyped and subjected to both static and dynamic testing. The force transducer based on a quartz capillary presented the lowest cross-sensitivity to temperature ($12\;mN{/}^{\circ }$C) and good accuracy (maximum measurement error of 65 mN/10 N) in a measurement of static forces. However, limited strength of the sensor is expected to prevent usage of the quartz capillary in small diameter needles. The concepts for a sensor based on an Invar capillary or a thin polyimide film proved a higher cross-sensitivity to temperature ($50\;mN{/}^{\circ }$C and $220\;mN{/}^{\circ }$C, respectively) and higher maximum measurement error (350 mN/10 N, 800 mN/10 N), comparable to those of FBG-based sensors reported in literature, but are likely to be more suitable for integration in very small biopsy needles.

## 1. Introduction

Needles are nowadays used for a wide variety of therapeutic as well as diagnostic medical procedures, as percutaneous (through the skin) needle insertion has proven to be an ideal method to gain invasive access to regions deep into the human body with minimal tissue damage [1,2]. As a result, minimally invasive needle insertion has become one of the most common procedures in medicine [2,3]. Regional anesthesia, biopsy, neurosurgery, deep brain stimulation, catheterization, ablation and brachytherapy are a few examples of complex procedures that rely heavily on the use of medical needles and accurate needle insertion [1,4,5]. New developments to improve needle insertion are of great interest due to the frequent occurrence of percutaneous needle insertions in healthcare [2]. One of the main developments of the last decennia in this growing field is technology enhanced needle insertion, such as image guidance and force feedback during perforation of the tissue [6]. Many recent academic studies related to needle insertion focus on tissue and tissue-tool-interaction models to model insertion, force feedback, robot assisted needle insertion devices, steerable needles and instrumented needles [2,7,8,9,10,11,12].

Accurate measurement of forces acting on a needle tip can be useful for multiple applications related to needle insertion, including research on tissue-tool interaction and (enhancing) force feedback. In present times a preference can be observed towards (fiber-)optical sensors for the measurement of forces on the tip of a needle. One of the main advantages of fiber-optic sensors is the potentially small size of the sensor as well as the required cabling [13]. Furthermore, fiber-optic sensors (to be precise, their optical sensing part) are inherently immune to electromagnetic interference, while electronic sensors are susceptible to electromagnetic interference to at least some degree. This is an important advantage in terms of medical applications, as it allows usage of the sensor in combination with RF-ablation and MRI scanners; environments with a need for force sensing at the tip of a needle (e.g., force feedback in robotic needle insertion devices [14,15]) but which are not optimal for electronic sensing systems. Several other advantages of fiber-optics sensors include (a) a fully dielectric working mechanism and hence no possibility of flow of electric current to the patient [13,16,17]; (b) biocompatibility and thus no predicament with invasive measurements and, finally; (c) the potential for multiplexing several fiber-optic sensors in a single optical fiber, allowing for a multidisciplinary diagnostic tool at the tip of a needle [13,17].

Despite the recent research activity, most force sensors for the measurement of axial forces on the tip of a needle discussed in literature, including optical sensors such as Fiber Bragg Gratings (FBGs), are unsuited for in vivo applications due to their large cross-sensitivity to temperature [12,18]. Therefore, the primary requirement for the design of our force sensor is a low cross-sensitivity to the temperature of the needle shaft. Secondly, the sensor should fit inside an 18 G needle and, more importantly, should be relatively easy to manufacture and assemble. Moreover, the sensor should be able to measure axial forces up to 10 N with a maximum measurement uncertainty of 500 mN/10 N [18,19], while the sensor is exposed to temperature variations between 17 ${}^{\circ }$C and 41 ${}^{\circ }$C.

To comply with the requirements of low cross-sensitivity to temperature and elementary fabrication it was chosen to design a sensor based on Fabry-Pérot interferometry [20,21]. The sensitive part of the Fabry-Pérot interferometer (FPI) can be fabricated on top of a cleaved optical fiber and can potentially be extremely short, thus making the FPI particularly suited to measure forces on a needle tip. Moreover, the cross-sensitivity for temperature of an FPI-based strain gage has been reported as low as 0.95 pm/${}^{\circ }$C, which is roughly a factor 90 lower than that of an FBG-based senor [22], indicating promising opportunities for FPI-based force sensors. It is estimated that the dimensions of the elastic element required to obtain a workable force range and sensitivity can be kept small, ensuring that the FPI sensor will fit in a very thin needle. Finally, FPI sensors are low cost, disposable and straight forward to fabricate and therefore seem the logical choice as a force sensor for harsh environments.

The goal of this study was to design a force sensor based on an FPI, incorporated in a needle tip, for the measurement of forces acting on the needle tip in axial direction of the needle. To realize this goal eight concepts for the force sensor were developed. Out of these eight concepts, the three most promising designs were simulated using finite element analysis. After selection of the most promising materials and production method, the three concepts were prototyped and subjected to both static and dynamic testing. On the basis of these tests a recommendation for further research will be given.

## 2. Working Principle and Readout of the Force Sensor

**Figure 1:**
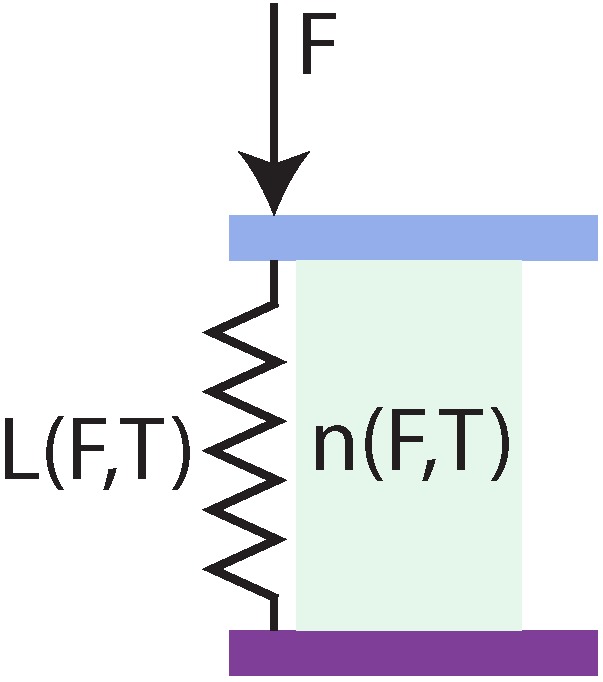
Simplified model of the Fabry-Pérot interferometer inside the force sensor, showing the mirrors of the FPI in purple and light blue with an FPI cavity medium in between with a refractive index *n* that depends both on temperature and stress inside the medium resulting from the force applied on the sensor. The mirrors are separated by an elastic element with distance *L* that depends on the temperature of the sensor and the force exerted on the elastic element.

**Figure 2:**
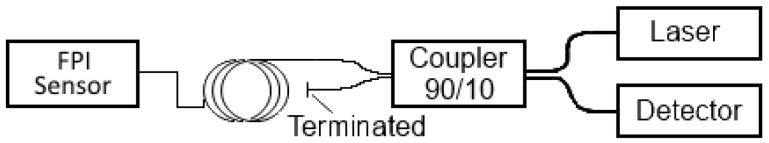
Schematic view of the readout system used to detect changes in the length of the elastic element in the FPI sensor. No light reflects at the terminated fiber end.

Figure 1 presents a simplified model of the heart of the force sensor, the Fabry-Pérot interferometer. The interferometer consists of an elastic element which is centred between two partially reflective mirrors and is fabricated on top of the cleaved end of an optical fiber. Light propagating trough the fiber partially reflects on the first mirror of the FPI and travels back into the fiber. The remainder of the light propagates through the elastic element, reflects on the second mirror and is ultimately collected in the optical fiber. The light waves propagating back to the light source interfere with each other and encode for the length of the elastic element. The back-propagating light is collected by a detector via a 90/10 fiber coupler (Mode hopping in the laser is minimized by introducing an isolator in the optical path), as demonstrated in Figure 2. When we neglect multiple reflections in the elastic element, we can describe the amplitude of the ideal interference signal in the photodiode by:

(1) $$W\left(d\right)=W_{0}\left[1+V\cos\left(\frac{4\pi L}{\lambda }+\varphi_{0}\right)\right]$$

where *L* is the length of the elastic element, $\varphi_{0}$ is a constant phase shift that only depends on the geometry of the probe, *λ* is the wavelength of the laser, and $W_{0}$ and *V* are the midpoint interference signal and the fringe visibility, respectively. The interference signal on the detector can be transformed to a linear readout of *L* following the mathematical derivation in [23].

A major requirement of the to-be-designed force sensor was a low intrinsic cross-sensitivity to temperature. FPIs are reported to have a particularly low sensitivity to temperature when the cavity is filled with air [24,25]. The elastic element, connecting the two mirrors, can be seen as a spring with compliance *c*, which carries the entire load on the force sensor and is assumed to compress according to Hooke’s law. The compliance of the spring is linearly dependent on the temperature of the sensor and independent of the exerted force:

(2) $$c=\frac{\partial L}{\partial F}=c_{0}+\frac{\partial c}{\partial T}T.$$

The change in length of the FPI cavity upon exertion of force is assumed to be equal to the change in length of the spring plus the change in geometrical length of the cavity due to expansion of the material connecting both mirrors. This geometrical change of cavity length is assumed to depend linearly on the temperature of the sensor and to be proportional to the coefficient of thermal expansion (CTE) of the spring’s material and the length of the FPI cavity (this definition implies that the length of the spring does not have to match the length of the FPI cavity). The length of the cavity is $L_{0}$ when no force is exerted on the sensor for a given temperature. The cavity length is thus:

(3) $$L=\left(L_{0}+\frac{\partial L}{\partial T}T+\left(c_{0}+\frac{\partial c}{\partial T}T\right)F\right).$$

The refractive index of the medium between the FPI mirrors is assumed to depend linearly on the temperature and the force exerted on the sensor (the latter will only be the case when the FPI cavity medium is a solid material). The refractive index is $n_{0}$ when no force is exerted on the sensor and the temperature of the sensor is ‘zero’. The refractive index is thus:

(4) $$n=\left(n_{0}+\frac{\partial n}{\partial T}T+\frac{\partial n}{\partial F}F\right).$$

The equation for the optical path difference (OPD) of an FPI is a function of the FPI cavity length *L* and the refractive index of the FPI cavity medium:

(5) OPD=2×n×L.

Summarizing all of the above, the OPD of the FPI of the force sensor as function of the force exerted on the force sensor and the temperature of the force sensor is thus:

(6) $$OPD\left(T,F\right)=2\times \left(n_{0}+\frac{\partial n}{\partial T}T+\frac{\partial n}{\partial F}F\right)\left(L_{0}+\frac{\partial L}{\partial T}T+\left(c_{0}+\frac{\partial c}{\partial T}T\right)F\right).$$

A change in OPD is only dependent on force or temperature. Cross-Sensitivity of the force sensor to temperature can occur in the form of a constant error and a modifying error. Change of the OPD as result of a change in temperature due to thermal expansion of the spring and a change in refractive index of the FPI cavity medium will result in a constant measurement error throughout the measurement range. The dependency of the Young modulus of the material of the spring on the temperature will on the other hand cause a modifying error on the force exerted on the sensor that will increase with increasing force. Assuming that the compliance of the spring does not significantly depend on temperature, the change in OPD can be described by:

(7) $$\Delta OPD_{linearized}=2\times \left(\frac{\partial n}{\partial T}\times L+2\times n\times CTE\times L\right)\Delta T+2\times \left(\frac{\partial n}{\partial F}\times L+n\times c\right)\Delta F$$

This is a reasonable assumption, as long as materials with a high glass transition temperature are selected for the spring (see Section 4). Equation (7) shows that the cross-sensitivity to temperature of an FPI used to measure force can be minimized by balancing the thermo-optic coefficient of the medium inside the FPI cavity and the CTE of the spring of the FPI. Both the thermo-optic coefficient and CTE should nonetheless be preferably as small as possible to minimize cross-sensitivity to temperature, as both material properties will likely never be sufficiently perfect in balance. Minimizing the FPI cavity length will also minimize the cross-sensitivity to temperature, as long as other parameters like the compliance of the spring remain constant. A reduction of the cavity length is limited by the manufacturing method and the minimum required length of the FPI cavity for good functionality. The cross-sensitivity to temperature can furthermore be minimized by maximizing the compliance of the elastic element of the FPI or maximizing the elasto-optic coefficient of the FPI cavity medium. This method of reducing the measurement error due to temperature disturbances is strongly limited by the range in which changes of OPD can be measured. The compliance of the elastic element can be increased by selecting an appropriate material with a low Young modulus for the elastic element and optimizing the dimensions and geometry of the elastic element of the FPI.

## 3. Concept Design and Fabrication

**Figure 3:**
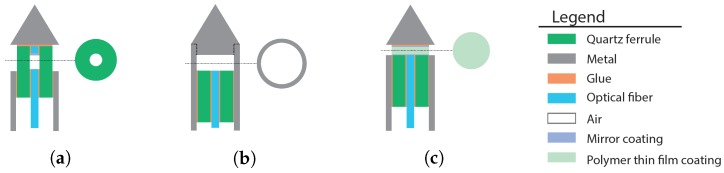
The three most promising concepts for a fiber-optic force sensor, based on Fabry-Pérot interferometry, in the tip of the stylet of a trocar needle to measure forces in axial direction on the needle tip, showing (**a**) the quartz capillary concept; (**b**) the Invar capillary concept; and (**c**) the thin flim concept. The illustrations of each concept show an axial cross-section of the stylet tip and a transverse cross-section of the stylet tip at locations containing an FPI cavity.

Several concepts for a FPI-based system to measure forces on the tip of the stylet of a needle, with a low cross-sensitivity to temperature, were generated based on an analysis of the requirements and suitable materials. In particular many combinations of different types of force-sensitive elements and placements of FPI sensors for force and temperature measurement were identified. The three most promising concepts, namely the quartz capillary concept, the Invar capillary concept and the polyimide film concept, are presented in Figure 3 and will be described in the next sections. We have selected two concepts with an air-filled cavity to minimize sensitivity to temperature and a column based spring of either quartz or Invar, based on yield strength, Young modulus and CTE. Furthermore, for comparison, one concept based on a polymer thin film that functions both as FP cavity medium and force sensitive element was selected. The concepts are demonstrated as prototypes, meaning that their design might not be ideal for the final application.

### 3.1. Quartz Capillary Concept

**Figure 4:**
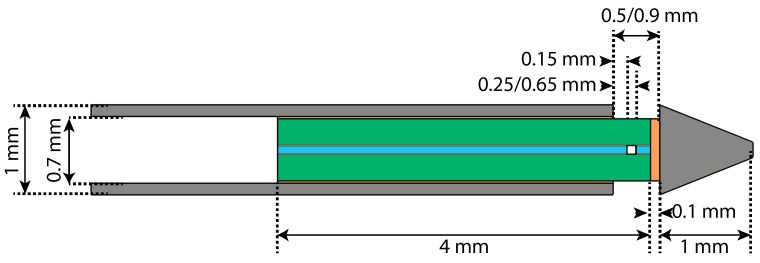
Cross-section of the geometry of the quartz capillary concept. The metal stylet tube and needle tip are shown in grey, the quartz ferrule in green, the glue layers in orange and the silica optical fiber in blue. The thickness of the glue layer used to fixate the optical fibers in the borehole of the quartz ferrule is enlarged for illustrative purposes. The FPI-cavity length was varied between 100 μm and 500 μm.

The sensing element of the quartz capillary concept (Figure 4) consists of a 6 mm long and 0.7 mm outer diameter clear fused quartz ferrule and two cleaved single mode optical fibers (SMF-28). The ferrule has a centred bore hole of 127±1 μm diameter with a 2 mm long tapered lead-in on one side of the ferrule. The prototype sensor was fabricated by glueing two fibers into the central bore hole of the quartz ferrule with a heat curable epoxy (EPO-TEK 353ND) (For higher mechanical and thermal stability the stripped fibers can be fused in the ferrule. For practical reasons this is neglected in the prototype). One of the fibers is connected to the FPI interrogation device, creating a Fabry-Pérot (FP) cavity between the facets of the cleaved fibers, while the other is sputter coated with a gold film (±50 nm) to prevent a second cavity. The force sensitive element of the quartz capillary concept thus consists of a hollow quartz column around the FP cavity, which is closed off from the environment by the ferrule and glue. Using a two component epoxy, the ferrule was subsequently glued into an stainless steel (AISI 304) stylet tube such that the sensitive part of the sensor still protrudes from the stylet. Finally, the needle tip was glued on top of the ferrule.

The cross-sensitivity to temperature of the quartz capillary concept is mainly dependent on the geometry of the column. However, a trade-off exists between the cross-sensitivity to temperature and the strength of the sensor, as a thin column will be much more susceptible to transverse stresses. During temperature fluctuations the expansion of the entire length of the column of the force sensitive element will result in a change of geometrical length of the FP cavity. However, the CTE of quartz nearly compensates the thermo-optic coefficient of air and matches the CTE of the silica fibers, hence the measurement error due to temperature disturbances, resulting from the thermal expansion of the quartz column and the thermo-optic coefficient of air (see Equation (7)), is expected to be lower than the maximum measurement uncertainty.

The brittle nature of the quartz glass column is the main weakness of the concept, but is not expected to hamper the performance in this study (see Section 4). Although the column structure was chosen to minimise the influence of transverse forces, a certain sensitivity towards the lateral bending force will remain imminent, unless the centre of the FPI cavity is located perfectly on the neutral axis of the needle. The cross-sensitivity can be approximated as the ratio of the stress in axial direction in the ferrule at the center of the FPI cavity per Newton transverse force and the stress per Newton axial force on the needle tip. The most important assumption of this approximation is that optical path of the light in the Fabry-Pérot cavity bends with the bending of the ferrule, which is not the case in practice. The calculated measurement error is therefore an underestimation, as the change in geometric length of the optical path through the FPI cavity due to bending will be larger than approximated. Furthermore, bending of the ferrule causes a wedge angle of the FPI mirrors which reflects light out of the FPI cavity, reducing visibility of the sensor particularly when the FPI cavity is long.

The ratio of the stress inside the ferrule per Newton transverse force and the stress per Newton axial force on the needle tip at the center of the FPI cavity can be decribed by:

(8) $$\frac{\sigma_{trans}}{\sigma_{ax}}=\frac{4\times L_{tip}\times x}{r^{2}}$$

where *x* is the eccentricity of the FPI from the neutral axis of the ferrule along the axis of the transverse force, *r* the radius of the ferrule and $L_{tip}$ the moment arm of the transverse force with the respect to the location of the center of the FPI. The cross-sensitivity to transverse force on the needle tip is according to Equation (8), approximately 42 mN per Newton transverse force per micron eccentricity of the FPI cavity. As a result of the low tolerance on the eccentricity of the boreholes of the ferrules used in our sensor, we expect the eccentricity in the sensor not to exceed 10 μm. The measurement error due to a 1 N transverse force on the needle tip is, therefore, estimated to be in the order of a few hundred milli-Newtons. Significantly reducing the sensitivity of the force sensor in the needle tip to transverse force on the needle tip is not straightforward, as it depends heavily on the precision of the manufacturing method.

### 3.2. Invar Capillary Concept

**Figure 5:**
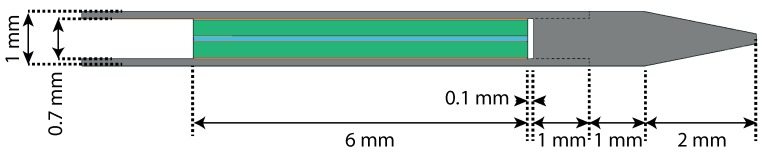
Geometry and dimensions of the Invar capillary concept, showing Invar parts in grey, a fused quartz ferrule in green, an optical fiber in blue and glue layers in orange. The press-fit connection between the needle tip and the stylet tube is shown as a dashed line. The aim for the FPI-cavity length was 100 μm.

Similarly to the fabrication of a sensor according the quartz capillary concept, the Invar capillary concept (Figure 5) consists of a cleaved single mode optical fiber, glued into the central bore hole of a quartz ferrule using a UV curable adhesive. However, this time the cleaved facet of the fiber is aligned with the facet of the ferrule. Next, the quartz ferrule with the integrated fiber is carefully inserted into an Invar capillary stylet tube until a calibrated depth and fixed with slowly curing glue. The insertion depth will determine the length of the FPI cavity. The end of a needle tip, also fabricated out of Invar, is polished to create a mirroring surface. The needle tip is then attached to the stylet tube by means of a press fit. An air filled FPI cavity will thus be created between the fiber-air transition at the end of the optical fiber and the polished surface of the Invar needle tip. The stylet tube will thus be used as an elastic column, creating the force sensitive element.

An advantage of the Invar capillary concept compared to the quartz capillary concept is the strength of the sensor. The metal stylet tube will carry practically all the tensile stress due to transverse forces on the needle tip and the brittle quartz ferrule will hardly be exposed to large tensile stresses. The cross-sensitivity to lateral forces is not expected to differ significantly from the quartz capillary concept, as the eccentricity still depends on the accuracy of the placement of the borehole (see Equation (8)). The cross-sensitivity to temperature of the FPI is expected to be higher than the quartz capillary concept, due to the slight mismatch in CTE of the quartz ferrule and the Invar stylet tube acting as the force sensitive element. Nonetheless, the required accuracy can be achieved when other sources of measurement errors do not contribute much to the total measurement error. Moreover, the relatively high cross-sensitivity to temperature could be compensated by introducing a second, temperature sensitive FPI, by filtering or by calibrating the sensor at body temperature.

There is unfortunately quite some chance that slip of the press fit connection of the needle tip, which also acts as second mirror of the force sensing FPI, will introduce an hysteresis error in the force sensor. Using glue instead of a press fit to attach the needle tip to the stylet tube could be a solution for this problem. Usage of glue is however not preferred, as there is a great risk that glue will flow into the FPI cavity that cannot be removed before the glue is cured. Furthermore, the usage of Invar, a ferromagnetic material, is a disadvantage of this concept, as it makes the needle unsuited for MRI-related applications.

### 3.3. Polyimide Film Concept

**Figure 6:**
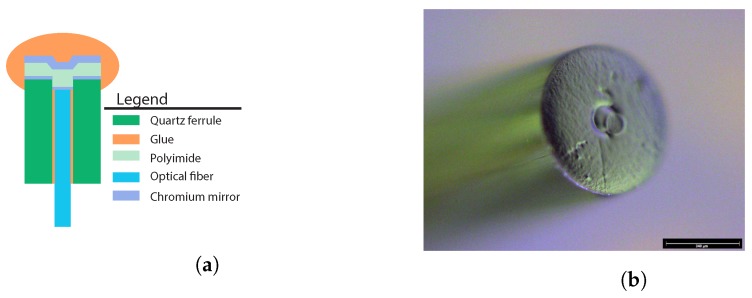
(**a**) Sketch of the cross-section of the thin-film concept. (**b**) Close up image of the proof-of-concept of the thin-film device, after deposition of the last 100 nm thick chromium layer. A small particle seems to be partially covering the optical fiber located in the center of the ferrule.

The two previously proposed concepts have FP cavities that can be strenuous to manufacture; during fabrication the challenge lies in precisely determining the length of the cavity, reproducing this cavity length for multiple sensors, and preventing that the air filled cavities are accidentally flooded with the glue that is used to assemble the sensor and the needle. The polyimide film concept therefore uses a polymer thin film as both FP cavity medium and force sensitive element, as illustrated in Figure 6. The length of such a cavity can be precisely controlled and reproduced with a thin film deposition method.

A sensor based on the parallel thin-film concept is fabricated fixing a cleaved single mode optical fiber in the bore hole of the quartz ferrule, analogous to the previously described concepts. A chromium film, which will act as the reference mirror of the force sensing FPI, is afterwards deposited by means of sputtering on the top facet of the quartz ferrule. A spin coated polyimide layer is subsequently deposited on top of the titanium-dioxide layer after treatment of the surface with an adhesion promoter. The elastic modulus of polyimide is expected to be hardly dependent on temperature thanks to its high glass transition temperature [26]. A thick chromium layer is finally deposited on top of the polymer layer by means of sputtering to serve as the second mirror of the FPI and prevent a double FP cavity. The needle tip can be glued on top of the ferrule and the ferrule can be glued into the stylet tube, identical to the quartz capillary concept. To increase strength and stability of the sensor, a polymer needle tip (or a metal needle tip with a polymer transition piece) can be attached on top of the stylet tube, a procedure that is unnecessarily complicated for the prototype phase. The sensor can be interrogated with interferometry by coupling the distal end of the fiber to the readout system.

Of the three presented concepts the polyimide film concept was expected to have the highest cross-sensitivity to temperature due to the large mismatch between the CTE of the polymer and that of the quartz substrate and due to the high thermo-optic coefficient of the polymer used for the combined FPI cavity medium and force sensitive element. The thermo-optic coefficient of polymers can be estimated with following equation, that uses the volumetric thermal expansion coefficient of the polymer to predict its thermo-optic coefficient [27]:

(9) $$\frac{dn}{dT}=-{\left(\rho \frac{dn}{d\rho }\right)}_{T}\times CTE_{vol}-{\left(\frac{dn}{dT}\right)}_{\rho }.$$

Disregarding the elasto-optic effect, the change in OPD of the FPI as function of force applied on the needle tip can be estimated with the following equation:

(10) $$\Delta OPD\left(\Delta F\right)=\frac{2\times n_{polyimide}\times L_{cavity}}{E_{polyimide}\times A_{ferrule}}\times \Delta F.$$

This equation is only valid for a thin film of which the substrate has identical mechanical properties as the polymer of the film itself and the stress distribution in the thin film due to the applied force is uniform. The change in OPD due to a change in temperature can be estimated by:

(11) $$\Delta OPD\left(\Delta T\right)=2\times \left({\frac{dn}{dT}}_{polyimide}+n_{polyimide}\times CTE_{polyimide}\right)\times L_{cavity}\times \Delta T,$$

when the thin film is deposited on a substrate with the same thermal and mechanical properties. The constant measurement error due to a change of temperature of 24 °C for a FPI made on a 0.7 mm diameter ferrule with a polyimide layer with a stiffness of 3.3 GPa, CTE of 32 μstrain/K and a layer thickness of 10 μm was estimated to be a 8 mN (marginally worse when the elasto-optic effect would have been taken into account). The low cross-sensitivity to temperature of the thin-film concept is the result of the influence of the CTE and the thermo-optic coefficient on the change in OPD of the FPI due to a change in temperature that nearly cancel each other out. This is however not the case when the polyimide film is deposited on a substrate with different mechanical and thermal properties than the polymer film.

It is therefore expected that the introduction of a second FPI sensor, solely dedicated to measuring temperature variations, might be beneficial to this concept. This temperature sensitive FP cavity can be positioned in a groove machined on the long edge of the ferrule. The polyimide film sensor is expected to be robust when a needle tip is moulded onto the stylet tube, but more fragile when the needle tip is glued. The FPI sensor is expected to be hardly sensitive to transverse forces, which can only result in a minimal shear of the polymer layer, thanks to the small cavity length.

## 4. Finite Element Analysis

Before prototyping of the three concepts a finite element analysis (FEA) of each concept was performed in order to assess the strength of the design and the sensitivity of the sensor to force. In addition, the influence of disturbances on the measurement uncertainty of the three designs were investigated. The FE simulations were performed with Solidworks and COMSOL Multiphysics. For all concepts, the bond line thickness of the glue, used to bond the ferrule to the needle tip and to the stylet tube, was assumed to be 25 μm. The Young modulus and the Poisson ratio of the glue, unless otherwise indicated, were assumed to be 0.5 GPa and 0.3, respectively.

### 4.1. Quartz Capillary Concept

A cross-section of the geometry of the axisymmetric FE-model of the quartz capillary concept is presented in Figure 4. FE-simulations (based on the modified Mohr-Coulomb criterion [28]) showed that the maximum equivalent stress due to a transverse load of 1 N on the needle tip was 47 MPa in the protruding quartz element. This is marginally lower than the reported maximum tensile stress of quartz glass (50 MPa). However, the maximum tensile stress of the quartz glass of the ferrule is expected to be at least a 2 fold higher as a result of very high surface quality, as was further supported by experiments in our lab. It was therefore decided that the limited strength of the quartz column was not a reason to discard the concept, particularly since transverse forces over 1 N are not expected. Further results of the FE-analysis are presented in Table 1.

Simulations were performed for sensors manufactured with NOA 68 UV curable adhesive or EPO-TEK 353ND epoxy to fixate the optical fiber in the ferrule, for Invar and metal stylet tubes and for a cavity length of 100 or 500 μm. For comparison, a glass fuse between the fiber and the ferrule is also taken into account. It can be observed, as expected, that the glue significantly determines the compliance of the force sensitive element, particularly when the compliant UV curable adhesive is used. A change in cavity length due to a 10 N load on the tip of at least 100 nm is preferred for a good visibility of the sensor. A relatively strong influence of the material of the stylet tube can be observed on the change in cavity length caused by temperature. The advantage of the Invar stylet tube was, however, not regarded as significant for this concept. The disadvantage on the measurement error of gluing the fiber in the ferrule with respect to fusing was non-existent according to our FE-simulations. The high compliance and high CTE of the UV curable glue, resulting in the highest change in OPD due to force and temperature, made the use of this adhesive unfavourable.

### 4.2. Invar Capillary Concept

A cross-section of the geometry of the Invar capillary FE-model is presented in Figure 5. The Invar capillary was not tested for the maximum equivalent stress due to a transverse load of 1 N. A collapse of the Invar capillary was not expected thanks to its high maximum tensile stress. The results of an extensive FE-analysis, taking into account the modulus, CTE and thickness of the epoxy used to bond the ferrule in the capillary, the CTE of the Invar capillary and the type of adhesive used to fix the fiber into the ferrule, is presented in Table 2. It was concluded that usage of a stiff epoxy with a medium CTE was preferred to bond the ferrule inside the Invar capillary. It can further be observed that the error due to cross-sensitivity to temperature does not significantly depend on the used type of adhesive. A change in FPI cavity length due to a 10 N axial force on the needle tip of 200–300 nm and a constant measurement error up to 1.5 N due to a temperature change of 24 °C were expected based on the FE-analysis.

### 4.3. Polyimide Film Concept

Modeling the Polyimide film concept with a FE-model showed a severe impact of the quartz glass substrate, with a high stiffness and a low CTE, on the cross-sensitivity of the concept to temperature. The Polyimide film (10 μm, 3.3 GPa, CTE 32 μstrain/K) was modelled to be deposited on a quartz ferrule housing an optical fiber. The change in geometrical FPI cavity length was defined as the change in thickness of the polyimide film along the axis of symmetry of the model. The elasto-optic effect was not taken into account.The change in cavity length due to a change in temperature was calculated based on the thermal expansion of the thin film along the axis of symmetry of the model and the change in refractive index of the polymer film. The force on the needle tip was modelled as a distributed load over the entire polymer film. The change in FPI cavity length due to a 10 N load is expected to be approximately 52 nm based on the FE-model (about equivalent to a change in length of 100 nm of an air-filled FPI cavity, due to the difference in refractive index). A change in FPI cavity length due to a 10 N force in the order of 79 nm would be anticipated had the thin polymer layer been deposited on a polyimide ferrule with identical mechanical properties as the polyimide film, which could be realized using plastic fibers.

The constant measurement error due to a 24 °C temperature change was expected to be approximately 2 N based on the FE-model. This high sensitivity to temperature is due to the low expansion coefficient of the quartz substrate that prevents the polyimide layer from expanding in lateral direction. The polyimide expands more in axial direction instead, which is not compensated by variation in the refractive index of the polyimide. The presence of the adhesive used to fix the fiber in the ferrule hardly effected the sensitivity of the FPI to either temperature or force. Finally, literature indicated quite some dependence of the Young’s modulus of polyimides on temperature (about 5% change in modulus for a temperature change from 20 °C to 40 °C, corresponding to a modifying error due to cross-sensitivity to temperature of about 0.5 N for a force of 10 N on the needle tip), despite a high glass transition temperature [29].

## 5. Experimental Methods

**Figure 7:**
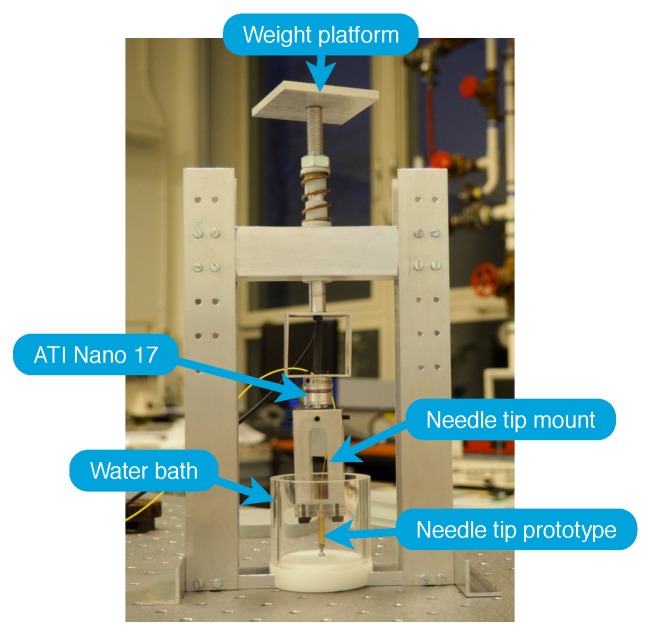
Calibration setup used to test prototypes of the needle tip containing an FPI-based force sensor.

The prototypes of the needle tips containing an FPI-based force sensor were tested using a custom made calibration setup shown in Figure 7. The calibration setup is placed on an active, air-stabilized optical table to minimize the effect of vibrations of the environment on the assessment of the resolution of the force sensors.

The calibration setup contains an ATI Nano 17 (ATI Industrial Automation, Apex, NC, USA) force sensor that serves as the reference sensor to determine the calibration force on the needle tip. Prototypes of the needle tips were mounted on the ATI Nano 17 sensor using a custom built needle tip mount. The ATI Nano 17 sensor was used to measure forces exerted in axial and transverse direction on the needle tip, as the calibration setup does not prevent accidental exertion of transverse forces on the needle tip.

During the calibration procedure axial forces were exerted on the needle tip both statically and dynamically. The force sensors were preloaded with the weight of the calibration setup, which was defined as a calibration force of 0 N. The needle tip was pressed onto the rigid bottom of a plastic water bath that could be filled with cold and hot water to test the cross-sensitivity of the prototype to temperature. A thermocouple was used to measure the water temperature. The accuracy of the thermocouple was in the order of 1 °C, which was determined by comparing readings of the thermocouple with readings of the precise thermometer of the IKA C-MAG HS 7 (IKA Werke GMBH, Staufen im Breisgau, Germany) heating plate used to heat water up to body temperature.

The force sensor of each prototype was tested for visibility prior to calibration and was interrogated using a commercial interferometer (OP1550 V2, Optics11, Amsterdam, The Netherlands). The wavelength was modulated around 1550 nm at 3 kHz with a modulation depth of approx. 50 pm. Data acquisition during the calibration procedures was performed using a custom application written in MATLAB.

### 5.1. Static Calibration

Axial static loads were exerted on the needle tip by placing well-determined weights on the platform of the calibration setup. Each prototype was first loaded 3 times for 60 s with the maximum calibration force (10 N). The calibration procedure was then performed by loading-and-unloading the prototype at least three times in small steps up to 10 N, during which readings of the reference force sensor and the prototype were obtained. A reading of the force sensors was obtained at least 5 s after changing the calibration force and was averaged over 1 s. Three times the standard deviation of the acquired 1 s long signal was defined as the resolution of the force sensor. Static calibration of prototypes was always performed at room temperature without submerging the prototype in water, as the water temperature could not be kept constant for a sufficiently long time. Static calibration took in practice at least 10 min per prototype. Due to this long measurement time significant drift of readings of the sensor, for example due to creep of the force sensitive element as a result of varying room temperature, may occur in the static calibration results. An average of at least three calibration cycles was taken for each concept to neutralise the drift.

### 5.2. Dynamic Calibration

Dynamic calibration of the force sensors was performed with the needle tip submerged in water and consisted of several 15 s long measurements. Arbitrary calibration forces were exerted on the prototype during these measurements by manually exerting force on the weight platform. The water was changed in between the measurements to vary the temperature of the environment. During the measurement the temperature of the water slowly changed to room temperature. Moreover, the transverse force on the needle tip varied strongly during dynamic calibration. This procedure can therefore give an impression of the performance of the needle tip force sensors under practical circumstances, where they can also be exposed to transverse forces and varying temperatures. The sensors were afterwards submerged in cold and hot water baths to evaluate the drift due to the change in temperature. The IKA heating plate was used to keep the hot water bath at constant temperature during these drift experiments.

### 5.3. Data Analysis

Interpolation functions, describing the readings of the optical detector of the sensor as function of the calibration force, were made by fitting an sinusoidal (in the case of small compression) or third order polynomial function (for larger compressions) to the data obtained during the static or dynamic calibration of the force sensor. The measurement error of prototypes was assessed as the difference between the calibration force according to the reference sensor and the force measured with the prototype, calculated from the reading of the FPI with the determined interpolation function. Errors can be made in the assessment of the measurement error of purely axial forces during static calibration when the transverse force on the needle tip varies during the calibration procedure. The estimated response of the FPI to transverse forces on the needle tip, derived from the ATI Nano 17, was therefore subtracted from the measured response of the FPI.

## 6. Results

In this section the results for static and dynamic calibration will be discussed for prototypes of all three concepts.

### 6.1. Quartz Capillary

The tested prototype had an FPI cavity length of roughly 500 μm and the ferrule protruded approximately 1.5 mm from the stylet tube (the FPI cavity was located as close as possible to the edge of the stylet tube). The change in FPI cavity length due to application of a load of 10 N on the needle tip was about 310 nm, as was predicted by the FE-simulations (Table 1).

**Figure 8:**
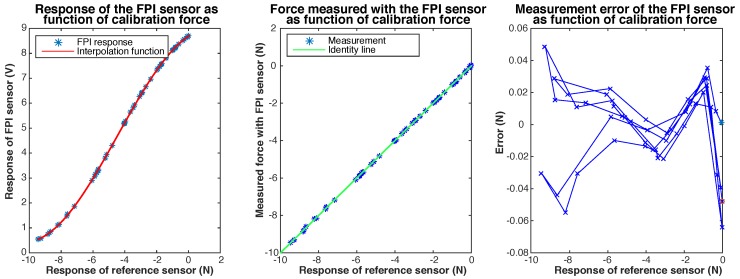
Results of static calibration of a prototype of the quartz capillary concept. The response of the FPI as function of the calibration force, together with a fitted sinusoidal interpolation function, is shown on the left. The center figure shows the force measured with the FPI, calculated using the interpolation function, as function of the calibration force and the indentity line (y=x) as a reference. The measurement error of the FPI sensor as function of the calibration force is shown on the right.

Results of static calibration of the prototype are shown in Figure 8.

The maximum observed measurement error during static calibration remains below 65 mN (without accounting for the resolution of the sensor). The largest measurement errors are observed at the limits of the measurement range. The resolution, as well as the distribution of noise, of the FPI-based sensor and the ATI Nano 17 sensor are found to be very similar.

**Figure 9:**
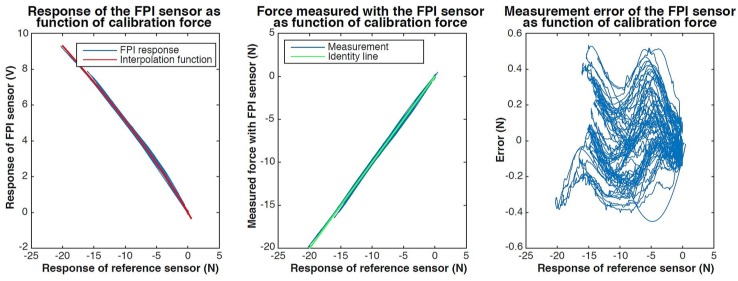
Calibration results of dynamic calibration with temperatures varying between 22 °C and 40° of the prototype of the quartz capillary concept. The response of the FPI as function of the calibration force, together with the fit, is shown on the left. The center figure shows the force measured with the FPI, calculated using the interpolation function, as function of the calibration force and the indentity line (y=x) as a reference. The measurement error of the FPI sensor as function of the calibration force is shown on the right.

**Figure 10:**
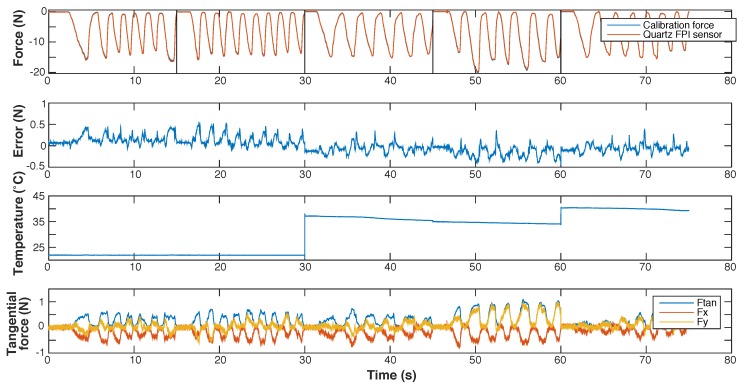
Results of dynamic calibration with temperatures varying between 22 °C and 40° of the prototype of the quartz capillary concept, shown as function of time. The graphs show, from top to bottom: (1) the calibration force and the force measured with the prototype, calculated using the interpolation function shown in Figure 9; (2) the measurement error of the prototype; (3) the temperature of the water bath in which the needle tip is submerged; (4) the transverse force on the needle tip, shown as the resultant transverse force (Ftan) and its two orthogonal components (Fx and Fy). The dotted lines in the top graph (1) indicate the 15 s long fragments of which the dynamic calibration dataset is composed.

In Figure 9 and Figure 10 we present the results of the dynamic calibration of the prototype. To test the temperature sensitivity of the sensor, the water temperature was increased stepwise from room temperature (22 °C) to the body temperature of a feverish patient (40 °C) during dynamic calibration. The maximum observed measurement error was 534 mN for a measurement range up to 20 N. The resolution of the prototype was estimated to be 9.5 mN, by analyzing a 1 s long measurement of the response of the prototype for a calibration force of 0 N. The resolution of the reference force sensor, the ATI Nano 17, was determined to be 2.7 mN. The maximum observed measurement error at room temperature, during dynamic calibration, was 388 mN.

Cross-sensitivity of the prototype to temperature was investigated by fitting separate linear interpolation functions to data obtained during dynamic calibration of the sensor at temperatures below 22.5 °C and data obtained at temperatures above 40 °C. Usage of the interpolation function determined for the response of the prototype at 40 °C while the needle tip is at room temperature (22.5 °C), would result in an error of varying between 190 mN and 240 mN within the measurement range (on average 215 mN) according to the fitted interpolation functions. This corresponds to a cross-sensitivity to temperature of $12\;mN{/}^{\circ }$C. The maximum resultant transverse force on the tip reached up to 1.1 N during dynamic calibration. The cross-sensitivity of the FPI to transverse forces was estimated to be 0.3–0.5 N/N axial force based on the regression model.

### 6.2. Invar Capillary

The Invar capillary prototype had an FPI cavity length of approximately 160 μm. The change in FPI cavity length due to a force of 10 N on the needle tip was approximately 700 nm.

**Figure 11:**
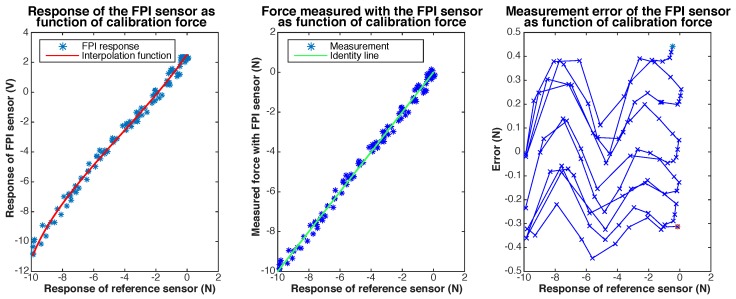
Results of static calibration of a prototype of the Invar capillary concept, not accounting for transverse force on the needle tip. The response of the FPI as function of the calibration force, together with a ploynomal fit, is shown on the left. The graph in the center shows the force measured with the FPI, calculated using the interpolation function, as function of the calibration force and the indentity line (y=x) as a reference. The measurement error of the FPI-based sensor as function of the calibration force is shown on the right.

The results of static calibration of the prototype are presented in Figure 11. Five instead of three calibration cycles were performed as the sensor seemed to suffer from significant drift, possibly due to viscoelastic creep of the glue. The maximum observed measurement error was 445 mN.

The maximum observed error of the FPI for the measurement of axial forces, after correction for variation in the resultant transverse force, is about 350 mN. The resolution of the prototype is estimated to be in the worst case, disregarding outliers, about 8 mN, which is about 2.5 times worse than the resolution of the ATI Nano 17 sensor. The resolution of the prototype is in the best case about equal to the resolution of the reference sensor.

**Figure 12:**
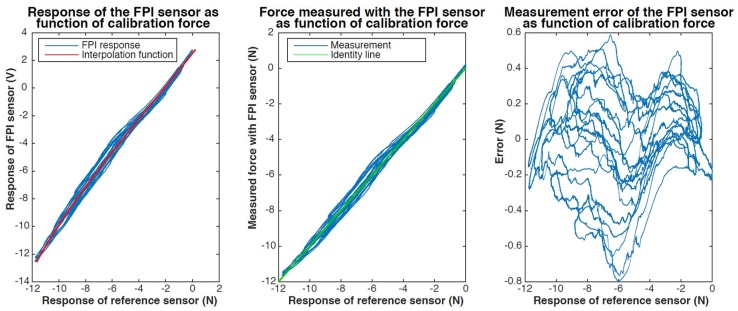
Results of dynamic calibration of a prototype of the Invar capillary concept. The response of the FPI as function of the calibration force, together with a fitted third order polynomial interpolation function, is shown on the left. The graph in the center shows the force measured with the FPI-based sensor, calculated using the interpolation function, as function of the calibration force and the indentity line (y=x) as a reference. The measurement error of the FPI-based sensor as function of the calibration force is shown on the right.

**Figure 13:**
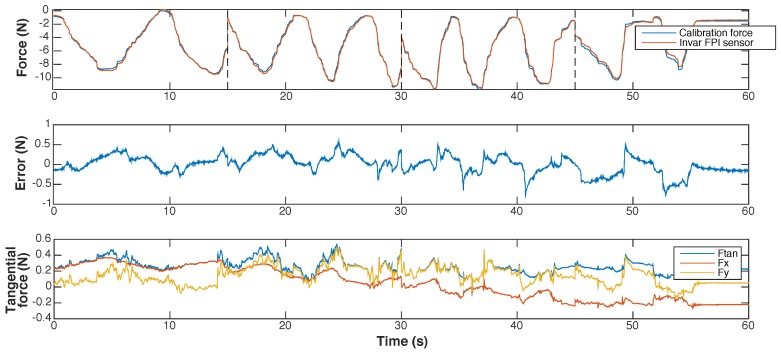
Results of dynamic calibration of a prototype of the Invar capillary concept at room temperature shown as function of time. The graphs present, from top to bottom: (1) the calibration force and the force measured with the prototype, calculated using the interpolation function shown in Figure 12; (2) the measurement error of the prototype; (3) the transverse force on the needle tip, shown as the resultant transverse force (Ftan) and its two orthogonal components (Fx and Fy). The dotted lines in the top graph (1) indicate the 15 s long fragments of which the dataset obtained during dynamic calibration is composed.

In Figure 12 and Figure 13 we present the results of dynamic calibration of the prototype of the Invar capillary concept at constant temperature. The Invar prototype demonstrated significant drift as a result of temperature variations and could therefore not be calibrated at fluctuating temperature.

The maximum observed measurement error during dynamic calibration using a 3rd order polynomial interpolation function was about 800 mN. The maximum resultant transverse force on the tip was 0.5 N during dynamic calibration. The cross-sensitivity of the FPI to transverse forces was estimated to be 0.6–0.9 N/N axial force based on the regression model.

After submerging the sensor in water of 40 °C, the prototype showed a continuous drift of 25 mN/s. An estimate for the cross-sensitivity to temperature could not be given as it was not possible to calibrate for the drift.

### 6.3. Thin Film

The FPI cavity length of the proof-of-concept was estimated to be in the order of 8.5 μm to 9.5 μm. The reflection of the FPI was less than expected, most likely caused by a dirt particle on the top of the fiber, which was observed under the microscope (Figure 6b). The sensitivity of the FPI to force was found to be around 30% of the predicted sensitivity in the FE-model. The change in FPI cavity length due to a force of 10 N on the sensor was approximately 16 nm.

**Figure 14:**
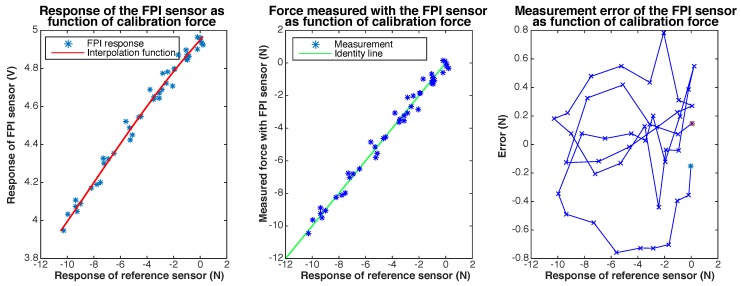
Results of static calibration of a proof-of-concept of the thin film concept. The response of the FPI as function of the calibration force, together with a fitted 3rd order polynomial interpolation function is shown on the left. The center figure shows the force measured with the FPI, calculated using the interpolation function, as function of the calibration force and the indentity line (y=x) as a reference. The measurement error of the FPI sensor as function of the calibration force is shown on the right.

Results of static calibration of the proof-of-concept, using a 3rd order polynomial interpolation function, are shown in Figure 14. The maximum observed measurement error within the measurement range is about 800 mN. The resolution of the proof-of-concept remains to be improved and slowly deteriorated during static calibration from 25 mN to 40 mN. This is significantly lower than the resolution of the reference sensor used during the calibration procedure (2.7 mN).

**Figure 15:**
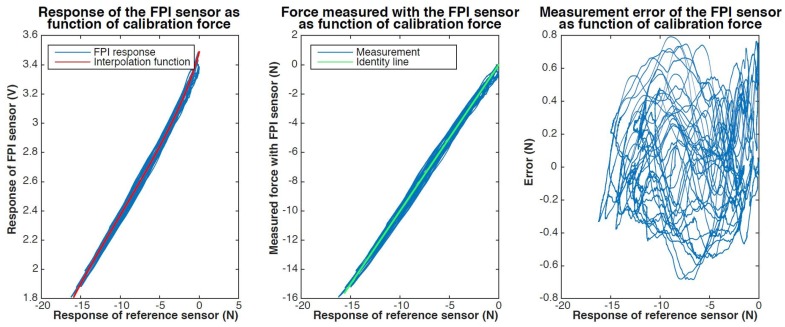
Results of dynamic calibration of a proof-of-concept of the thin-film concept at room temperature. The response of the FPI as function of the calibration force, together with third order polynomial interpolation function, is shown in the figure on the left. The figure in the middle shows the force measured with the proof-of-concept, calculated using the interpolation function, as function of the calibration force and the indentity line (y=x) as a reference. The measurement error of the proof-of-concept as function of the calibration force is shown on the right.

**Figure 16:**
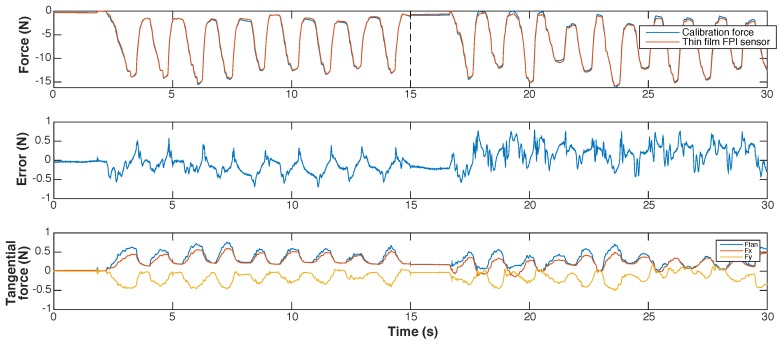
Calibration results of dynamic calibration of a proof-of-concept of the thin-film concept at room temperature, shown as function of time. The graphs show, from top to bottom: (1) the calibration force and the force measured with the proof-of-concept, calculated using the interpolation function shown in Figure 15; (2) the measurement error of the proof-of-concept; (3) the transverse force on the needle tip, shown as the resultant transverse force (Ftan) and its two orthogonal components (Fx and Fy). The dotted lines in the top graph (1) indicate the 15 s long fragments of which the dataset obtained during dynamic calibration is composed.

Results of dynamic calibration at constant temperature of the thin film proof-of-concept, using a 3rd order polynomial interpolation function, are shown in Figure 15 and Figure 16. The maximum observed measurement error within the shown dataset, consisting out of two separate 15 s long measurements, is on average 800 mN.

Calibration of the thin film sensor at fluctuating temperatures proved laborious due to similar drifts as found with the Invar capillary prototype. The measurement error due to cross-sensitivity to temperature is for a temperature change from about 23 °C to 39 °C estimated to be on average 3.5 N when linear interpolation functions are used to describe the response of the FPI. This results in a cross-sensitivity to temperature of $220\;mN{/}^{\circ }$C. The thin film sensor was rather insensitive to transverse forces in its current form; the maximum resultant transverse force did not exceed 0.5 N during dynamic calibration. The cross-sensitivity of the FPI to transverse forces was estimated to be around 0.03 N/N axial force.

## 7. Discussion

Three concepts were presented for integrating an FPI sensor into the tip of a needle in order to measure axial forces. Prototypes of these concepts were analysed in an FE-environment, built and calibrated, both statically and dynamically, and their performance was evaluated in terms of accuracy for axial forces, sensitivity for lateral forces and temperature cross-sensitivity.

Although the calibration setup as described in Section 5 was not designed to separate axial from transverse loads on the needle tip, the ATI Nano 17 reference sensor was able to detect the transverse loads applied on the needle tip. Where possible, the measured load by the FPI sensor was corrected for the transverse load given by the ATI Nano 17. Small differences in alignment between the reference sensor and the FPI sensor might have led to an over- or under correction and thereby influenced the calculated sensitivity of the FPI sensor to axial forces. The variation of transverse forces on the needle tip was minimized as much as possible by using masses on the weight platform during static calibration. However, small unintentional variations of the transverse force, especially during dynamic calibration, were unavoidable.

The calibration results of the quartz capillary concept agreed very well with the FE-simulations. Both the expected force sensitivity and temperature sensitivity were demonstrated by the prototype. The measured cross-sensitivity to transverse forces matched the theoretical calculation discussed in Section 3. The quartz column did not show any sign of damage after calibration, despite transverse forces up to 1.1 N during the procedure.

The force response of the Invar capillary concept agreed with FE-analysis. Regrettably, due to heavy drift of the FPI sensor after a temperature change, we were not able to report on the temperature cross-sensitivity of the concept. It is, however, safe to assume that the concept as such is unfit for use in an environment with fluctuating temperature. The observed drift is most likely caused by the mismatch in CTE between quartz and Invar the multitude of glue joints used in the current prototype. Unlike the quartz based prototype, the FPI cavity of the Invar concept exists of two different materials and entails a large mechanical loop including several glue joints. Replacing these joints with fuses in a future prototype may improve the temperature sensitivity of the design drastically. Out of the two capillary designs, the Invar concept was expected to be the most resistant to lateral forces. However, dynamic calibration resulted in a larger cross-sensitivity to lateral forces for the Invar prototype. This decreased performance can be described to an underestimation of the effect of the several glue joints in the Invar concept. An additional downside of the Invar concept is the loss of potential MRI compatibility.

The force sensitivity of the thin film concept was only 30% of the sensitivity expected from the FE-analysis. This can be partly explained by a slightly thinner polyimde layer as well as a possible underestimation of the Young modulus due to neglection of the elasto-optic effect. Fabrication of the FPI by means of thin film deposition proved to be an advantage. The controllability of the sensitivity of the sensor during manufacturing makes the thin film concept better suited for usage in combination with interferometry compared to the other two concepts. Despite this fabrication advantage, the sensitivity of the thin film prototype tested in this study was high, as predicted by the FE-analysis. The simulations show that when the polyimide layer is deposited on a substrate with a similar CTE, the temperature sensitivity can be reduced to as low as 330 μN/°C. The main drawback of a polyimide elastic element is the sensitivity of its compliance to the temperature of the sensor [29]. In this case, incorporation of an additional temperature sensing FPI inside the needle tip might still be required when no polymer for the thin film can be found with a constant elastic modulus within working temperature range. The sensitivity to transverse stresses was low during static and dynamic calibration. However, addition of a needle tip to the prototype might increase the transverse force per Newton axial force. Therefore, the cross-sensitivity to transverse forces may be underestimated in this study.

The FEA did not account for undefined properties of the glue that could results in measurement errors, such as creep or viscoelastic behaviour of the glue or a temperature dependent compliance. These properties made the use of a compliant glue less attractive, as they would be more evident. To avoid creep and drift as much as possible, non compliant, glass-like glues were selected for FEA. Ultimately the FE-model was able to predict the behaviour of the various prototypes adequately.

Calibration of the quartz capillary concept yielded an order of magnitude improvement in measurement uncertainty of static axial forces compared to FBG-based sensors designed earlier at the TU Delft, approaching the accuracy and resolution of the ATI NANO 17. The intrinsic low cross-sensitivity to temperature of the prototype was nearly two orders of magnitude better than FBG-based needle tip force sensors shown in literature [12]. The exceptional low cross-sensitivity of the prototype comes at the cost of a quite fragile needle tip. More reliable strength tests are required to determine the safety margin of the design, although FE-simulations showed that the current design could be sufficiently strong for integration in 18 G needles.

Marginally visible in the response of the quartz capillary prototype, but more evident with the Invar capillary sensor, was the effect of the glue joints in the sensor and between the sensor and the needle shaft. Measurements of the FPI sensor seemed to lag slightly behind those of the reference sensor during dynamic calibration, resulting in a hysteresis loop. A correlation between sudden large changes of the calibration force and sudden changes in measurement error can be observed in the calibration results shown in Figure 13. The correlation becomes more distinctive in the last two 15 s segments of the dataset obtained during dynamic calibration, where changes in axial calibration force occur more abruptly. This can be an indication of a viscoelastic response of the force sensitive element and is assumed to caused by the viscous component of the glue in the joint. Prototypes that used softer glues demonstrated this effect more clearly, further strengthening our assumptions. Eliminating the glue by fusing the readout fiber in the ferrule and, in case of the Invar-based prototype, replacing the glue joints with fuses would be preferable but too complicated for a first prototype.

A significant sensitivity of all prototypes to transverse force could be observed, predominantly during the dynamic calibration procedure. Using the current manufacturing method and materials it proved impossible to fabricate a sensor that is perfectly located on the neutral axis of the needle. Therefore, for future prototypes, it is recommended to implement a biaxial or, preferably, a triaxial FPI sensor. The implementation of a triaxial FPI sensor could, specifically in the the case of the Invar-based prototype, increase the accuracy by separating the transverse forces from the axial forces.

The proposed prototypes proved to be able to detect small force variations while maintaining a low cross sensitivity to temperature. While the current prototypes were optimized for an application with a 18 G needle, all optical FPI force sensors are likely to be suitable for integration in very small biopsy needles. Their ability to measure small variations in the needle insertion force can be employed for needle targeting in future research.

## 8. Conclusions

A study of different concepts for a small fiber-optic force sensor based on Fabry-Pérot interferometry to measure forces in axial direction on the tip of a needle was performed. The goal was to design a sensor with a low cross-sensitivity to temperature of the needle tip, an adequate resolution to measure axial loads in the desired range and the sensor was furthermore preferred to be easily manufacturable and, possibly, MRI compatible. Three different concepts for a force sensor in the needle tip with distinct advantages were investigated in more detail. The force sensor based on a quartz capillary resulted in a very low cross-sensitivity to temperature and high axial force resolution, but might be hampered by the fragility of the quartz element. The Invar-based force sensor demonstrated good mechanical strength and decent force resolution but suffered from a higher cross-sensitivity to temperature and transverse forces. The thin film sensor showed great promise for future research, although the current prototype was not able to reach the desired resolution and temperature sensitivity. The small size of this sensor, enabling a tri-axial readout of the force, and the possible integration with plastic fibers, however, makes this concept the most promising for continued research.

## Figures and Tables

**Table 1:** Results of the FE-analyse of the sensitivity of the FPI of the quartz capillary concept to force and temperature. The first column shows the type bond used to fix the optical fiber in the ferrule (UV curable NOA 68, epoxy EPO-TEK 353ND or fused glass), the material used for the stylet tube and needle tip and the cavity length. The second column shows the change in geometrical cavity length (OPD) due to application of a 10 N load on the needle tip. The third column shows the change in cavity length (OPD) as a response to an increase in temperature of 24 °C, accounting for the CTE of materials used in the needle tip and the thermo-optic coefficient of air. The last column shows the expected measurement error due to cross-sensitivity to temperature in mN/°C.

Glue Type, Stylet Metal, Cavity Length (μm)	Change in OPD due to 10 N Axial Force (nm)	Change in OPD due to 24 °C Temp. Incr. (nm)	Error due to Cross-Sensitivity to Temp. (mN/°C)
UV cur, RVS, 100	−243.4	7.2	−12.5
Epoxy, RVS, 100	−103.7	2.8	−11.25
Fused, RVS, 100	−95.3	2.9	−12.5
UV cur, Invar, 100	−243.7	−1.7	2.91
Epoxy, Invar, 100	−103.8	−1.1	4.58
Fused, Invar, 100	−95.4	−0.7	2.91
UV cur, RVS, 500	−390.6	4.3	−4.58
Expoxy, RVS, 500	−251,4	0.37	−0.42

**Table 2:** Results of FE-analysis of the Invar capillary concept, investigating the sensitivity of the force sensing FPI in the needle tip to force and temperature, as function of the CTE of Invar, the thickness of the epoxy layer between the ferrule and Invar capillary, the modulus of elasticity and CTE of the epoxy and the type of glue used to fix the optical fiber into the ferrule. The needle tip was assumed to be attached rigidly, without a glue layer, to the stylet tube. The FE-model accounts for the elastic modulus and CTE of used materials and the thermo-optic coefficient of air. The sensitivity to force is shown as the change in geometrical cavity length due to an applied axial force of 10 N on the needle tip. The sensitivity to temperature is shown as change in cavity length due to a temperature change of 24 °C. The last three columns show the expected measurement error due to cross-sensitivity to temperature in mN/°C.

Configuration: ${\mathrm{CTE}}_{\mathrm{Invar}}$ Modulus Glue (GPa), Glue Borehole	Glue Layer Thickness (μm)	Change in OPD due to 10 N (nm)	Change in OPD due to 24 °C Temp. Incr. (nm)			Error due to Cross-Sensitivity to Temp. (mN/°C)		
			${\mathrm{CTE}}_{\mathrm{epoxy}}$: 100 μstrain/K	${\mathrm{CTE}}_{\mathrm{epoxy}}$: 50 μstrain/K	${\mathrm{CTE}}_{\mathrm{epoxy}}$: 10 μstrain/K	${\mathrm{CTE}}_{\mathrm{epoxy}}$: 100 μstrain/K	${\mathrm{CTE}}_{\mathrm{epoxy}}$: 50 μstrain/K	${\mathrm{CTE}}_{\mathrm{epoxy}}$: 10 μstrain/K
1.25, 0.5, UV cur	25	−297	−2.30	8.30	16.7	3.30	11.7	23.3
	50	−433	−12.9	5.50	20.1	12.5	5.40	19.2
2, 0.5, UV cur	25	−297	20.3	30.8	39.2	28.3	43.3	55.0
	50	−433	14.9	33.3	48.0	14.2	32.1	46.3
1.25, 0.5, Epoxy	25	−293	−3.20	8.00	17.0	4.60	11.3	24.2
	50	−430	−14.1	5.10	20.5	13.8	5.00	20.0
2, 0.5, Epoxy	25	−293	19.2	30.4	39.3	27.5	43.3	55.8
	50	−430	13.6	32.8	48.1	13.3	31.7	46.7
1.25, 3.5, UV cur	25	−185	−10.5	−0.30	7.80	23.8	0.80	17.5
	50	−246	−29.9	−9.00	7.70	50.8	15.4	12.9
2, 3.5, UV cur	25	−185	2.50	12.7	20.9	5.80	28.8	47.1
	50	−246	14.9	6.00	22.7	25.4	10.0	38.3
1.25, 3.5, Epoxy	25	−178	−13.3	−1.50	7.90	31.3	3.30	18.3
	50	−239	−34.3	−10.0	7.60	60.0	17.5	13.3
2, 3.5, Epoxy	25	−178	−0.60	11.2	20.6	1.30	26.3	48.3
	50	−239	19.6	3.80	22.4	34.2	6.70	39.2

## Abbreviations

The following abbreviations are used in this manuscript:

FPI	Fabry-Pérot interferometer
FP	Fabry-Pérot
FBG	Fiber bragg grating
MRI	Magnetic resonance imaging
RF	Radio frequency
CTE	Coefficient of thermal expansion
FEA	Finite elemelent analysis
OPD	Optical path difference
